# Assessment of Vaccine Hesitancy to a COVID-19 Vaccine in Cameroonian Adults and Its Global Implication

**DOI:** 10.3390/vaccines9020175

**Published:** 2021-02-19

**Authors:** Jerome Nyhalah Dinga, Leontine Kouemou Sinda, Vincent P. K. Titanji

**Affiliations:** 1 Biotechnology Unit, Faculty of Science, University of Buea, P.O. Box 63, Buea, Cameroon; 2 Department of Microbiology and Parasitology, Faulty of Science, University of Buea, P.O. Box 63, Buea, Cameroon; leontinesinda@yahoo.com; 3 Faculty of Science, Engineering and Technology, Cameroon Christian University Institute, P.O. Box 5, Bali, Cameroon; vpk.titanji@yahoo.com

**Keywords:** COVID-19 pandemic, vaccine hesitancy, vaccine acceptance, clinical trials, Cameroon

## Abstract

Since the outbreak of COVID-19 in December 2019, no global consensus treatment has been developed and generally accepted for the disease. However, eradicating the disease will require a safe and efficacious vaccine. In order to prepare for the eventual development of a safe and efficacious COVID-19 vaccine and to enhance its uptake, it is imperative to assess vaccine hesitancy in Cameroonians. After obtaining ethical clearance from the Institutional Review Board of the University of Buea, a questionnaire was administered (May–August 2020) to consenting adults either online or in person. A qualitative thematic analysis was done to analyze the participants’ answers to the open questions. A deductive approach was used, that is, the codes and patterns according to the World Health Organization (WHO) Strategic Advisory Group of Experts (SAGE) Working Group Matrix of Determinants of vaccine hesitancy. The number of consenting adult Cameroonians who completed the questionnaire were 2512 (Two thousand five hundred and twelve). Vaccine hesitancy to a COVID-19 vaccine was 84.6% in Cameroonians. Using the WHO recommended Matrix of Determinant of Vaccine hesitancy, the most prominent determinants observed in this study were: Communication and Media Environment, Perception of pharmaceutical industry, Reliability and/or source of vaccine and cost. Most Cameroonians agree that even though there are benefits of a clinical trial, they will prefer it should be done out of the continent and involving African scientists for eventual acceptance and uptake. The concerns of safety, efficacy and confidence has to be addressed using a Public Engagement approach if a COVID-19 vaccine has to be administered successfully in Africa or Cameroon specifically. Since this study was carried out following WHO standards, its result can be compared to those of other studies carried out in different cultural settings using similar standards.

## 1. Introduction

As of 17th August 2020, Cameroon had registered 18,599 cases of COVID-19 with 16,540 recoveries and 406 deaths. This gives a recovery rate of 88.9% and a with an overall death rate of 2.1% [1]. However, recent trends show that new cases of infection continue to emerge despite the control measures put in place by the World Health Organization (WHO) and the Ministry of Public Health in Cameroon. It is becoming evident that an efficacious vaccine would be required to put COVID-19 under control and eventually eradicate it. There are currently 138 candidate vaccines in preclinical evaluation and 29 candidate vaccines in clinical evaluation making a total of 167 vaccine candidates under development [2]. However, three COVID-19 vaccines have been developed but only the Comirnaty COVID-19 mRNA vaccine has received WHO Emergency Use Listing Procedure/Prequalification (WHO EUL/PQ) authorization. This made the Pfizer/BioNTech vaccine the first to receive emergency validation from the WHO since the outbreak began a year ago [3]. Hence, in order to be able to use these vaccines to eradicate the disease, reluctance to accept them by the population has to be reduced to the barest minimum.

**Figure 1:**
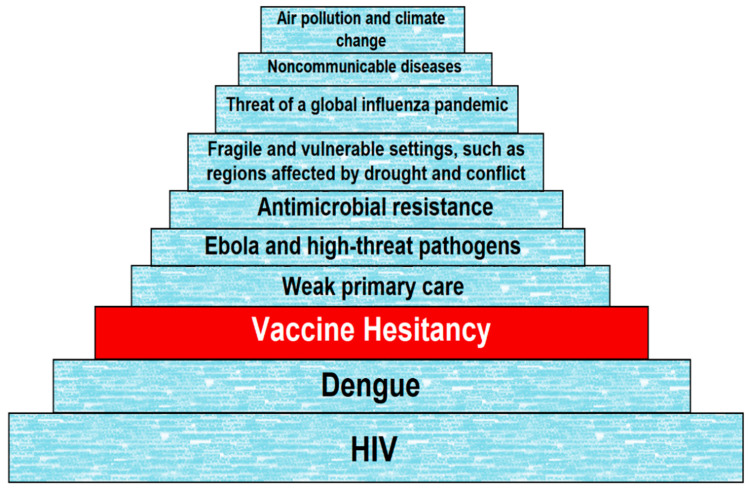
Top ten (10) threats to global health in 2019 according to the World Health Organization (WHO).

Vaccine hesitancy (VH) has been defined as “a delay in acceptance or refusal of vaccines despite availability of vaccine services” according to the World Health Organization (WHO) Strategic Advisory Group of Experts (SAGE) Working Group on VH [4]. VH has become the focus of growing concern and attention worldwide, despite overwhelming evidence of the importance of vaccines [5]. This is increasingly affecting the rate at which immunization regimes are effectively implemented. Since 2014, the number of countries reporting VH has been on a steady rise [6]. Only 14 countries out of 194 (about 7%) reported no VH according to a WHO/UNICEF Joint Reporting Form data from 2015–2017 [6], although an even lower number of seven (07) countries was reported in 2017 [7]. Research shows that VH is rising, resulting in alarming figures on disease outbreaks reported globally. This led the WHO to announce that, as of 2019, VH is one of the top ten threats to global health (Figure 1) [8].

Apparently, there is no single cause of VH because many factors come into play. Important drivers of VH include: perception that vaccines are not beneficial, pain and needle fear, concern about the safety of vaccines or distrust of the pharmaceutical industry in the implementation of vaccination programs [9,10,11]. “Fake news” or negative information about vaccination online and in social media is also an important cause of VH. In fact, many studies have shown that the equivocal nature of anti-vaccination information on the Internet contributes to an increase in VH [12,13,14,15]. Another frequently identified cause of VH is lack of knowledge about vaccines [16,17]. As a matter of fact, socioeconomic status and education are related to vaccine acceptance but not in the same way as they are related to health conditions or adherence to public health recommendations. On the contrary, increased VH has been associated with both high and low socioeconomic status as well as high and low education, highlighting the complex array of interrelated factors at play [10].

It has been shown that vaccine behaviors are complex, like most health behaviors and knowledge is only one of many determinants of vaccination decisions [9,18,19]. The three Cs model (confidence, complacency and convenience) indicates three key interrelated causes of VH [4]. With the advent of the COVID-19 pandemic and with the numerous vaccine candidates under development, it is imperative that an eventual efficacious vaccine should have an excellent uptake worldwide and Cameroon specifically. Here we try to identify the prevalence and determinants of a COVID-19 vaccine VH in Cameroonians.

## 2. Materials and Methods

### 2.1. Cameroon Demographics and Health System

Cameroon is a human mosaic full of over 200 ethnic groups and many national languages. Its population is estimated to have risen to over 24 million in 2019. With a surface area of 475,650 square km it gives a population density of 51 inhabitants per square km. The population is made of 48% young people below the age of 15 while 3.5% are aged 65 and above. The country has an annual growth rate of 2.6% [20].

As of 2016, Cameroon’s health map shows 1800 health areas and 189 health districts in 10 regions, including approximately 5166 public and private health facilities spread throughout the national territory. Access to health services in the country in 2016 was estimated to be 2.19 health facilities per 10,000 inhabitants. The health service facilities are organized into seven categories: general hospitals, central hospitals, regional hospitals, district hospitals, district medical centers, Integrated health centres and ambulatory health centres [21].

### 2.2. Study Design

A cross-sectional survey was carried out with Cameroonians living in the country or abroad from May to August 2020. This cross-sectional survey was done to encompass descriptive, quantitative and qualitative analysis to ascertain prevalence of VH.

The descriptive and quantitative parts aimed to establish the prevalence of VH amongst Cameroonians living at home or abroad. The qualitative part involved using the Matrix of Determinants for VH designed by WHO SAGE, in accordance with their recommendations [22]. This was done to assess reasons people gave to justify their hesitance while qualitative thematic analysis (QTA) was used to ensure the validity and reliability in detecting hesitancy across various cultural settings and permit global comparisons. We also assessed Cameroonians’ perception of a clinical trial.

### 2.3. Sample Size

The Raosoft sample size calculator [23] was used to ensure the recruitment of a sufficiently large number of participants in order to obtain statistically significant data, given the chosen of margin error (5%), confidence interval (95%) and population size (26,000,000 individuals). This gave a sample size of 385 participants.

### 2.4. Data Collection

After explaining the purpose of the study, assured about confidentiality and anonymity of the information requested, oral or written consent was obtained. Adults 18 years of age and above were then asked to fill a questionnaire (in French or English) on basic demographic characteristics, a COVID-19 vaccine hesitancy and vaccine clinical trials. The questionnaire was also designed such that expectations, benefits and concerns of a COVID-19 vaccine could be expressed and recorded. Some questions were added to conceal the real purpose of the survey in order to get unbiased responses. Questionnaires were administered either in person or online using Google Forms through Tweeter, Facebook messenger, pages and groups and WhatsApp groups.

The prevalence of VH to a COVID-19 vaccine was gotten by considering the answer to closed question 12 (Appendix A). A qualitative thematic analysis (QTA) was done (August/September 2020) to analyze the study participants’ answers to the open questions. A deductive approach was used, that is, the codes and patterns according to the SAGE WG Matrix of Determinants (MxDt) of VH (Table 1).

This method was chosen to ensure that the results are relevant worldwide. The approach developed by Braun and Clarke [24], used a combination of latent and semantic coding approaches to grasp both explicit and implicit content embedded within the data.

The following procedures were done by two separate researchers.

1. Familiarization with the MxDt of VH and the data.

2. The researchers then calibrate their understanding of how the dataset related to the 21 items of the SAGE MxDt.

3. After preliminary training, the two researchers identified 3697 statements that are directly linked to at least one of the 21 items. The binary methody (item present in the statement: 1/item absent from the statement: 0) was then used tothen independently mapped the 3697 statements directly against the 21 items of the Matrix, as previously reported [25].

4. Inter-coder reliability, using “ReCal2 0.1 (Alpha)” [26], an online utility which is compatible with Excel and computes reliability coefficients for ordinal, nominal or ratio-level data, was calculated for each of the 21 items, across the 3697 statements, using percent agreement and Cohen’s Kappa.

The results for “percent agreement” are between 0 and 1, where “0” implies the authors coding did not perform better than if they had been working at random, while “1” implies perfect agreement between the researchers. Negative results mean the researchers did worse than if they would accidentally. The cut-off values for Kappa were selected based on a method described by Hallgreen [27].

- from 0.0 to 0.2 = slight agreement
- 0.21 to 0.40 = fair agreement
- 0.41 to 0.60 = moderate agreement
- 0.61 to 0.80 = substantial agreement
- 0.81 to 1.00 = near perfect or perfect agreement.

### 2.5. Ethical Statement

Ethical clearance for this study was obtained from the Institutional Review Board of the Faculty of Science, University of Buea; Ref: 2020/1220-06/UB/SG/IRB/FHS. After explaining the purpose of the study and consent obtained (either written or verbal), a questionnaire was administered either in person or online using Google Forms.

### 2.6. Statistical Analysis

Data was analyzed using Microsoft excel and ReCal2 0.1 (Alpha). IBM SPSS Statistics 20 (IBM Corporation, Chicago, IL, USA) was used to calculate the 95% confidence interval and the significant differences amongst the 21 Items of the Matrix of Determinants of VH. *p* < 0.05 was considered statistically significant.

## 3. Results

### 3.1. Demographic Information

**Figure 2:**
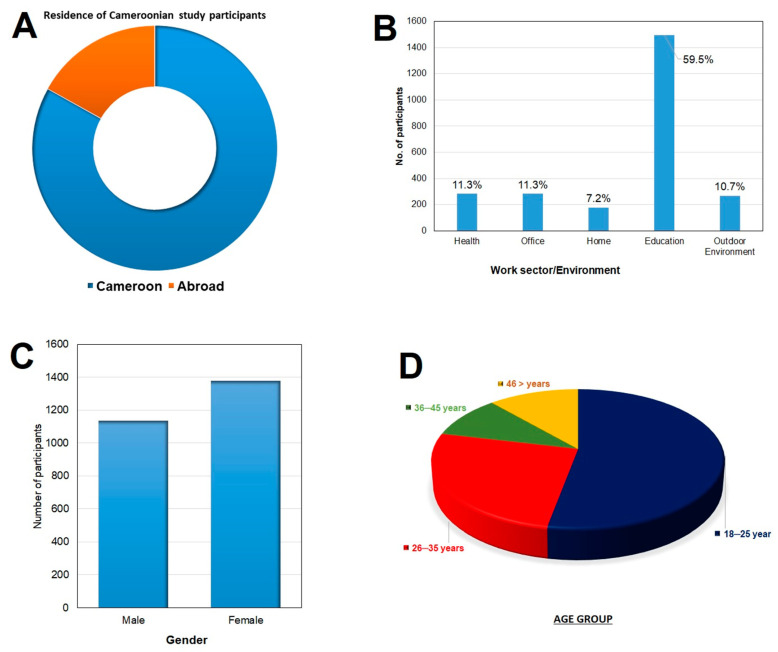
Demographic information of study participants. (**A**) most of the participants were residing in Cameroon while 17% of them lived out of the country. (**B**) Those working in the education sector constituted more than half of the participants. (**C**) shows the percentage males and females (**D**) shows the age of the participants with a majority of them being between the ages of 18–25.

The number of Cameroonians gave their consent and filled a questionnaire was 2512 (Two thousand five hundred and twelve). Of the 2512 participants, 2086 (83%) were residing in the national territory while 426 (17%) were living in the diaspora (Figure 2A). Participants from all the ten regions of Cameroon took part in this study. Most of them worked in the education sector and the least were working at home (Figure 2B). They were made up of more females 1378 (54.9%) than males 1134 (45.1%) (Figure 2C) and were mostly of the age group 18–25 years (Figure 2D).

### 3.2. Vaccine Hesitancy to a COVID-19 Vaccine

The number of the study participants who said they will need more information, do not know or will not take a COVID-19 vaccine stood at 2124 giving a VH prevalence of 84.6% (95% CI 83.1–85.9%). Those who said they will either need more information or they do not know or will refuse the vaccine were grouped into the vaccine hesitancy category.

### 3.3. Qualitative Thematic Analysis (QTA)

Table 2 presents the inter-rater reliability measures computed for each item of the MxDt. It shows the degree of agreement between the two researchers in coding the data. Except for Item 6 (which was not mentioned), no other Item had a score below 0.9 indicating the high level of agreement between the two. The large sample size of the study also created a condition to have a more frequent repetition of these items by the responders, hence the high degree of agreement. Additionally, item 6 was not mentioned once by either researcher, while those statements labelled “not codable” reflected comments that were not specific and ideas outside the matrix. The coding guide is found in Appendix B.

### 3.4. Contextual Influences

#### 3.4.1. Item 1 “Communication and Media Environment”

This item was frequently raised by participants to explain the reluctance to accept a COVID-19 vaccines (*p* < 0.001, when compared to Item 19 (Table 3)). Of particular interest here, is the role of Internet and the emergent role of social media such as Facebook, where it is known that sharing of anecdotal experiences, anti-vaccines campaigns and science denigrating content proliferate. This issue appears to be raised across the narratives of both COVID-19 vaccine hesitant and non-hesitant participants: participant 30 “Facebook”, participant 439 “confusing information circulating on social media” participant 2033 “anti-vaccine campaigns saying Africans should not accept a COVID-19 vaccine”, participant 984 “I’ve seen on TV that Africans don’t need a COVID-19 vaccine since there are herbal cures for the disease”.

#### 3.4.2. Item 7 “Perception of the Pharmaceutical Industry”

The issue of conflicts of interests and the respect for ethics by the pharmaceutical industry was raised by mostly COVID-19 vaccine hesitant individuals (*p* < 0.001, when compared to the non-hesitant group (Table 3)). They believed financial gains by these companies superseded the public health interest of the population. A few examples illustrate these negative attitudes:

- Participant 2285 stated, “I don’t think these companies really care about our health”.
- Participant 438 opined “Vaccine companies will send suboptimal vaccines to Africa”,
- Participant 2421 affirmed, “They take advantage of the political system to come and test vaccines and other products in Africa that they would not otherwise test in their own country and hence they will not respect standard ethical procedures”.

#### 3.4.3. Item 18 “Reliability and/or Source of Vaccine”

This item was present in an overwhelming majority (86.1%) of the statements (*p* < 0.001, when compared to Item 19 (Table 3)). They worried about the quality of the vaccine distributed or sent to Africa in general and Cameroon specifically.

- Participant 942 replied “why would they label drugs and vaccines for human use as “Not for use in USA and EU?”
- Participant 1985 wondered, “the vaccine sent to Africa will be of poor standards”,
- Participant 1469 declared, “I will only accept a COVID-19 vaccine from a reliable organization that has been tested and proven effective”.

#### 3.4.4. Item 20 “Cost”

Cost was also present in the majority of the statements 56% (*p* < 0.001, when compared to Item 19 (Table 3)) saying a COVID-19 vaccine should be made free for everyone while some people said it should be made free only to kids, the elderly, people with comorbidities and those working in hospitals.

The other items that showed up prominently among the responders include; Item 10 Knowledge/awareness (*p* < 0.001), Item 14 Risk/benefits (epidemiological and scientific evidence) (*p* < 0.001) and Item 16 Mode of administration (*p* < 0.001), All Items were compared to Item 19 since it is the item that appeared the least number of times (non-zero number) amongst the statements.

### 3.5. Perception of a COVID-19 Vaccine Clinical Trial

#### 3.5.1. Proposed Venue of a Clinical Trial

**Figure 3:**
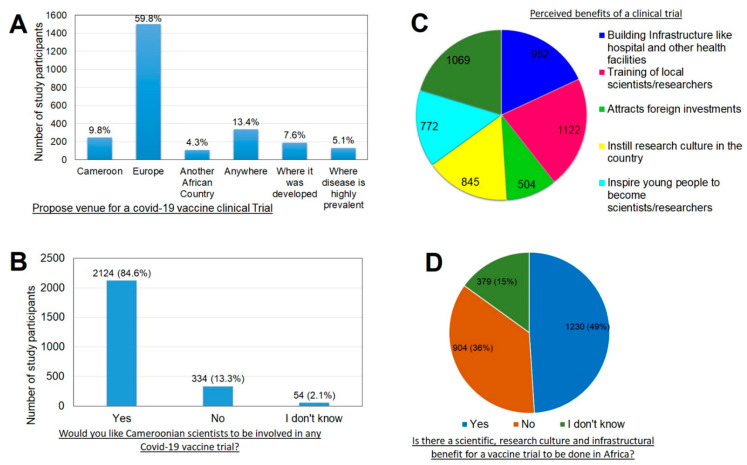
Cameroonian perception of a COVID-19 vaccine clinical trial. (**A**) proposed venues of a COVID-19 vaccine clinical trial. (**B**) opinions with respect to involvement of Cameroonian scientists in a COVID-19 vaccine clinical trial. (**C**) shows what Cameroonians think will be benefits of a well conducted clinical trial. (**D**) shows the percentage of the study participants who believe there are benefits/or not of a well-done clinical trial.

When asked where they think a COVID-19 vaccine clinical trial should take place, most Cameroonians proposed that it should be done in Europe (*p* < 0.001) with a small fraction saying it should be carried out where the vaccine was developed or where the disease is most prevalent (Figure 3A). Participant 398 proposed, “In Europe since they were hit hard by the pandemic,” participant 1212 “First trial in the country that manufactures/discovers the vaccine”.

#### 3.5.2. Involving Cameroonian Scientists

On the idea of involving Cameroonian scientists in the development of a COVID-19 vaccine, an overwhelming majority (*p* < 0.001) suggested that Cameroonian scientists should be involved. However, while some said they should be involved right from conception, others said they should be on board if they possess the skills and knowledge to do so. Some also said Cameroon should develop its own capacity to develop and produce a COVID-19 vaccine (Figure 3B). Participant 722 “Cameroonian scientists should be involved from conception”, Participant 884 “why not develop our own capacity to develop and produce a COVID-19 vaccine”?

#### 3.5.3. Knowledge of the Benefits of a Clinical Trial

Cameroonians were asked if there are any benefits of carrying out a COVID-19 vaccine clinical trial in Cameroon and most of them agreed there will be benefits especially in the area of training of local scientists/researchers and bringing about new drugs, vaccines and different ways of treatment (Figure 3C) (*p* < 0.001). Most Cameroonians agree that a well-executed clinical trial could assist in instilling a research culture in the country with infrastructural benefits (Figure 3D) (*p* < 0.001).

## 4. Discussion

Since the first diagnosed case of COVID-19 in December 2019, the number of new infections and fatalities continue to rise in some countries leading to a pandemic that is yet to be put under control. The World Health Organization (WHO) is yet to confirm and endorse a treatment of the disease and it is now a general consensus that the spread of the disease can be stopped by strict application of the barrier measures while eradicating the disease will require a vaccine. However, for the approved vaccines to achieve its intended goal, VH has to be reduced to under 30%–25%. It is therefore imperative to assess VH and its determinants locally in order to strategize and achieve significant vaccine acceptance. Here we report the first results of VH among Cameroonians using WHO SAGE recommendations.

It has been shown that a major source of access to health-related information is the Internet [28] and Social media in particular [29]. This has also been used by activists to carry out campaigns against the scientific institutions [30]. Studying the Internet content has also shown that future public health interventions need not only focus on the benefits of COVID-19 vaccination but, more so, on understanding the type of doubts around COVID-19 VH issue in order to enhance communication strategies [31].

Another item of the Matrix of Determinant of VH that came up strongly in this study is “Reliability and/or source of vaccine”. This shows the level of confidence and trust issues Cameroonians will have with a COVID-19 vaccine. Comments from participants show that they are really concerned about who is producing the vaccine and what will be the true intention of administering these vaccines to Cameroonians.

The next most frequent item raised by the participants was “Perception of pharmaceutical industry”. This seems to be expected giving the fact that pharmaceutical industries will be directly involved in producing and distributing a COVID-19 vaccine. From the comments of the participants, there seems to be a deep mistrust of pharmaceutical industries, especially those in the Western countries and in China. Respondents have the impression that the companies are interested only in making profit instead of thinking of the health of the population. It has been shown that people turn to be worried about risks produced by people or institutions they do not trust [31].

This study shows that Cameroonians will like Cameroonians or African scientists to be involved in the development of a COVID-19 vaccine. This again has to do with the level of trust they have in foreign institutions and pharmaceutical companies when it comes to a COVID-19 vaccine. The assumption here is that seeing one of theirs in the research team developing a vaccine will increase confidence. 

It is well known that travelling abroad broadens your horizon and makes you more knowledgeable [32] and so Cameroonians at home will usually turn to their relatives/friends in the diaspora for pieces of advice on critical issues. Hence, it was imperative to access VH of a COVID-19 vaccine in this set of Cameroonians. The opinions expressed by them followed the same trend as those back at home even though the tone was more objective and had less conspiracy-like tone. The issues raised were that of safety, efficacy and confidence.

Involving African scientists in general and Cameroonian scientists specifically, in the development of a COVID-19 vaccine will greatly enhance its uptake. This could be achieved by empowering these scientists through capacity building, infrastructure and funds to carry independent research in a consortium.

As concerns carrying out clinical trials, Cameroonians, especially those in the diaspora agreed that there are many benefits for such to be carried out in Cameroon including; infrastructure, capacity building, attracting foreign investments, instilling research culture and inspiring young people to become scientists. However, it was emphasized that this can only be achieved if the trials are carried out to the highest standards and respecting WHO recommended rules and regulations for a human clinical trial. This fear could be reflected in the fact that most of the Cameroonian respondents at home suggested that a COVID-19 vaccine clinical trial should be done either in Europe, where the vaccine was developed or where the disease is most prevalent.

The high prevalence of VH in Cameroon is worrisome and indicates a low appetite to have the vaccine and hence a low COVID-19 vaccine uptake. Therefore, a lot of measures have to be put in place to reduce the VH value to around 30% in order to achieve herd immunity to COVID-19 infection in Cameroonians.

Our projection is that the observations seen in the present study reflects the scenario one will encounter in other sub-Saharan countries. With the use of the WHO recommendations in assessing VH in a population, the results of the present study can be compared to that of other studies using the same approach in different geographical and cultural settings around the world.

Hence, community/public engagement will be the ultimate approach to significantly increase vaccine acceptance to a COVID-19 vaccine in Africans in general and Cameroonians specifically.

### 4.1. Strengths of The Study

To our knowledge, this is the first study in Cameroon to ever use the tools developed by the WHO to monitor vaccine hesitancy. It will bring a valuable contribution to public health strategies to enhance vaccine uptake.

The selection of study participants was not biased as people of all age groups, gender, those who regularly see medical providers/those who don’t, different economic and educational background took part in the study. The questionnaire was administered online and as well as in person to those who are not literate or do not have access to the internet.

The total number of participants was far larger than the calculated sample size of 385. The number of persons admitted in this study was large enough to permit declarations with 99% confidence level. This also permitted a high Cohen’s Kappa score hence preventing disagreement between the coders to weigh more on the score.

With responders from all the ten Regions of Cameroon as well as Cameroonians living out of the country, the data provide a nationally representative result.

This is a pilot study whose outcome could be used to properly design and execute a survey to determine VH for other vaccines.

### 4.2. Limitations of The Study

The questions were not pilot-tested as recommended in SAGE’s report from 2014 [24]. A pilot test could assist researchers reflect and alter the question sequence accordingly [33].

Some studies used electronic data extraction from the data and then mapped against the Matrix [34], but in the present study we used the deductive thematic analysis to map the statements of participants’ answers to matched the SAGE WG MxDt of vaccine hesitancy.

People of the older generation who are more vulnerable to the disease did not respond much to the survey probably due to limited access to online participation. Furthermore, the Cameroonian population is skewed towards the younger population with the result that the 18–25 years’ range is truly representative of the overall adult population.

## Figures and Tables

**Table 1:** Matrix of Determinants (MxDt) of vaccine hesitancy (VH).

Contextual influences	1	Communication & media environment
	2	Influential leaders, immune
	3	Historical influences
	4	Religion/culture/gender/socio-economic
	5	Politics/policies
	6	Geographical barriers
	7	Perception of pharmaceutical industry
Individual and group influences	8	Personal, family, and/or community member experience with vaccination, including pain
	9	Beliefs, attitudes
	10	Knowledge/awareness
	11	Health system and providers-trust and personal experience
	12	Risk/benefits (perceived, heuristics)
	13	Immunization as a social norm
Vaccine/vaccination specific issues	14	Risk/benefits (epidemiological and scientific evidence)
	15	Introduction of a new vaccine
	16	Mode of administration
	17	Design of vaccination program
	18	Reliability and/or source of vaccine
	19	Vaccination schedule
	20	Costs
	21	Strength of recommendation

**Table 2:** Inter-rater reliability measures for the items of the Matrix Determinant of Vaccine Hesitancy.

Item	Percent Agreement	Scott’s Pi	Cohen’s Kappa	Krippendorff’s Alpha	No. of Agreements	No. of Disagreements	No. Cases	No. of Decisions
Item 1	99.84	0.996	0.996	0.996	3691	6	3697	7394
Item 2	99.78	0.966	0.967	0.967	3689	8	3697	7394
Item 3	100	1	1	1	3697	0	3697	7394
Item 4	99.92	0.998	0.998	0.998	3694	3	3697	7394
Item 5	99.84	0.978	0.978	0.978	3691	6	3697	7394
Item 6	99.97	−0.001	0	0.000	3696	1	3697	7394
Item 7	99.59	0.988	0.988	0.988	3682	15	3697	7394
Item 8	100	1	1	1	3697	0	3697	7394
Item 9	100	1	1	1	3697	0	3697	7394
Item 10	99.89	0.997	0.998	0.998	3693	4	3697	7394
Item 11	99.95	0.994	0.994	0.994	3695	2	3697	7394
Item 12	99.92	0.996	0.996	0.996	3694	3	3697	7394
Item 13	100	1	1	1	3697	0	3697	7394
Item 14	99.76	0.995	0.995	0.995	3688	9	3697	7394
Item 15	100	1	1	1	3697	0	3697	7394
Item 16	99.65	0.992	0.992	0.992	3684	13	3697	7394
Item 17	100	1	1	1	3697	0	3697	7394
Item 18	99.24	0.969	0.969	0.969	3669	28	3697	7394
Item 19	100	1	1	1	3697	0	3697	7394
Item 20	99.62	0.992	0.992	0.992	3683	14	3697	7394
Item 21	100	1	1	1	3697	0	3697	7394

**Table 3:** Summary of how the study participants’ answers to some of the open questions are related to the determinants of vaccine hesitancy in the model developed by SAGE WG.

SAGE WG Vaccine Hesitancy Model ^a^					
Contextual Influences		Individual and Group Influences		Vaccine and Vaccination-Specific Issues	
Item	Iterations	Item	Iterations	Item	Iterations
1	**2356**	8	80	14	**1314**
2	120	9	214	15	208
3	30	10	**1362**	16	**1338**
4	974	11	170	17	22
5	138	12	380	18	3184
6	0	13	30	19	2 **^b^**
7	**2878**			20	**2072**
				21	120
TOTAL	6494 (38.2%)	TOTAL	2236 (13.2%)	TOTAL	8260 (48.6%)

**^a^** Total statement: 3697. SAGE WG: Strategic Advisory Group of Experts Working Group. **^b^** for statistical analysis, all the other Items were compared to Item 19 since it is the item that appeared the least number of times (non-zero number) amongst the statements. Boldface indicates statistical significance (*p* < 0.001).

## Data Availability

The data presented in this study are available in the article.

## Appendix A. Questionnaire

1. **Where do you live?**
  ۝Cameroon ۝Rest of the World
2. **Town:** ………………………………
3. **Gender:**۝Female ۝Male
4. **Age:**۝ 18–25 years ۝26–35 years ۝36–45 years ۝above 46 years
5. **Profession:** ……………………………………
6. **Which of the following best describes your working environment?**
  ۝Hospital ۝Office ۝Home ۝School ۝Outdoor environment
7. **Is there a vaccine against COVID-19?**
  ۝Yes ۝No ۝I don’t know? ۝Others: ……………………………………………….
8. **Have you heard information about the COVID-19 vaccines in development?**
  ۝Yes ۝No ۝Others: ….
9. **Where did you hear about this information?**
  ۝Social Media ۝Government radio TV ۝Newspapers ۝Internet
  ۝My surroundings ۝Others: ….
10. **What type of information from RELIABLE sources would you like to have on COVID-19 vaccines?**
  ۝Administration route ۝Dosage ۝Producer ۝Country of origin
  ۝Others: ……………………………………………….
11. **Should the COVID-19 vaccine be paid or given free of charge?**
  ۝Only free for children and elders ۝Free for everybody
  ۝Only free for health personnel ۝Paid for everybody ۝I don’t know
  ۝Others: …………………………………………………………
12. **Would you agree to receive a COVID-19 vaccine?**
  ۝Yes ۝No ۝I will need more information
  ۝I don’t know
13. **Through which channel would you like to receive all valid information on a COVID-19 vaccine?**
  ۝In a book ۝In a newspaper ۝On Radio/TV
  ۝Internet/Social media ۝Others: …………………………..
14. **Where would you like that a covid19 vaccine trial be carried out?**
  ۝In Cameroon ۝In another African country
  ۝In Europe
  ۝Others: …………………………..
15. **Would you like Cameroonian scientists to be involved in any COVID-19 vaccine trial?**
  ۝Yes ۝No ۝ I don’t know
16. **What are the benefits of well-done clinical trial?**
  ۝Building Infrastructure like hospital and other health facilities
  ۝Training of local scientists/researchers
  ۝Attracts foreign investments by international companies
  ۝Instill research culture in the country
  ۝Inspire young people to become scientists/researchers
  ۝brings about new drugs, vaccines and different ways of treatment
  ۝Others: ………………………………………..
17. **Is there a scientific, research culture and infrastructure benefit for a vaccine trial to be done in Africa?**
  ۝Yes ۝No ۝ I don’t know

## Appendix B

Coding rules/guide used by researchers for 21 items of the MxDt of VH using study participants’ answers to questions 8, 10, 11, 12, and 13 of the Questionnaire (Appendix A).

**Table:**

Words/Ideas/Phrase/Pattern Found in the Answer of the Study Participants to the Selected Questions	Item of MxDt Attributed to
Fake news, specific/unspecifc statement to serious side reaction of absurd or serious nature	1
Information from the media about the effects of vaccines	
Misinformation or conspiracy-like tone information about the health system	
Concerns about bad or misinformation	
Misinformation or concerns about bad information	2
Concerns about the interest and intentions of the health system	
Information on past vaccination campaigns and their outcome	3
Answers related to religion, culture and gender	4
Government policies, political in nature, community perception of vaccination	5
Related to geographical barriers	6
Distance from vaccination site	
Trust in the health care personnel	7
Fake news, specific/unspecifc statement to serious side reaction of absurd or serious nature	
Giving consent	
Little or no information about adverse effects	
Little or no information about manufacturer	
No precise additional information	
Personal experience or any community member of vaccination	8
Misinformation or concerns about bad information	9
Serious side effects/reactions	
“afraid of” or “fear”	
Correct statements about vaccination	10
Failure to be informed or lack of information	
Serious side effects/reactions	
Concerns about misinformation or failure to inform oneself	
No precise additional information	11
Misinformation or conspiracy-like tone information about the health system	
Concerns about the interest and intentions of the health system	
Issue of trust when it comes to vaccines	
Giving consent	
“afraid of” or “fear”	12
Correct statements about vaccination	
Safety concerns	
Little or no information about adverse effects	
Risks and benefits	
Social norm	13
Community participation	
Necessity of vaccine	
Concerns about misinformation or failure to inform oneself	
Concerns about misinformation or failure to inform oneself	14
“afraid of” or “fear”	
Safety concerns and efficiency	
True statement or trust associated with vaccination	
Lack of information	
Little or no information about adverse effects	
inform about about content	
failure to inform oneself	
Lack of information of health systems or vaccine manufacturers	
Necessity of vaccines	
Little or no information about adverse effects	
Route of administration	
Old vs new vaccines	15
Route of administration	16
Misinformation or conspiracy-like tone information about the health system	17
Lack of information of health systems	
Reputation/misinformation about vaccination programs	
Lack of information of health systems	18
Availability of vaccines	
Lack of information on vaccine manufacturers	
Health issues that can lead to delay in vaccination	19
timing	
Distance from vaccination site	20
Availability of vaccines	
Low income	
Little or no information about adverse effects	21
Lack of information of health systems	
Lack of information on vaccine manufacturers	
Lack of information	
Giving consent	
Confidence in health care personnel	
Misinformation or conspiracy-like tone information about the health system	
